# Gastrocnemius Medialis Architectural Properties in Flexibility Trained and Not Trained Child Female Athletes: A Pilot Study

**DOI:** 10.3390/sports8030029

**Published:** 2020-03-04

**Authors:** Ioli Panidi, Gregory C. Bogdanis, Vasiliki Gaspari, Polyxeni Spiliopoulou, Anastasia Donti, Gerasimos Terzis, Olyvia Donti

**Affiliations:** Sports Performance Laboratory, School of Physical Education & Sport Science, National and Kapodistrian University of Athens, 17237 Athens, Greece; ipanidi@phed.uoa.gr (I.P.); vgaspari@phed.uoa.gr (V.G.); spipolyxeni@phed.uoa.gr (P.S.); adonti@phed.uoa.gr (A.D.); gterzis@phed.uoa.gr (G.T.)

**Keywords:** youth, fascicle length, muscle thickness, maturation, stretching exercises, musculotendinous junction, ultrasound

## Abstract

Gastrocnemius medialis (GM) architecture and ankle angle were compared between flexibility trained (n = 10) and not trained (n = 6) female athletes, aged 8–10 years. Ankle angle, fascicle length, pennation angle and muscle thickness were measured at the mid-belly and the distal part of GM, at rest and at the end of one min of static stretching. Flexibility trained (FT) and not trained athletes (FNT) had similar fascicle length at the medial (4.19 ± 0.37 vs. 4.24 ± 0.54 cm, respectively, *p* = 0.841) and the distal part of GM (4.25 ± 0.35 vs. 4.18 ± 0.65 cm, respectively, *p* = 0.780), similar pennation angles, and muscle thickness (*p* > 0.216), and larger ankle angle at rest (120.9 ± 4.2 vs. 110.9 ± 5.8°, respectively, *p* = 0.001). During stretching, FT displayed greater fascicle elongation compared to FNT at the medial (+1.67 ± 0.37 vs. +1.28 ± 0.22 cm, respectively, *p* = 0.048) and the distal part (+1.84 ± 0.67 vs. +0.97 ± 0.97 cm, respectively, *p* = 0.013), larger change in joint angle and muscle tendon junction displacement (MTJ) (*p* < 0.001). Muscle thickness was similar in both groups (*p* > 0.053). Ankle dorsiflexion angle significantly correlated with fascicle elongation at the distal part of GM (*r* = −0.638, *p* < 0.01) and MTJ displacement (*r* = −0.610, *p* < 0.05). Collectively, FT had greater fascicle elongation at the medial and distal part of GM and greater MTJ displacement during stretching than FNT of similar age.

## 1. Introduction

Triceps-surae (gastrocnemius medialis, gastrocnemius lateralis, and soleus) muscles architecture is an important functional characteristic in athletes, patients, and the elderly [1,2], as these muscles are prime movers of the ankle joint during locomotion [3,4]. Longer and less pennate gastrocnemius muscle fascicles increase muscle shortening velocity and thus power output [5] while pennation angle and muscle thickness are positively correlated with muscle force production [6,7].

Maturational growth, from infant to adult, and mechanical stimuli alter triceps-surae muscle architecture [8,9]. During growth, muscles are continuously stretched due to skeletal development [10], but data on gastrocnemius architectural properties in developing children are limited [8,10,11]. Moreover, it is largely unknown how gastrocnemius muscles architecture is related to functional properties in youth athletes [8] although athletes participating in sports like gymnastics, figure skating or ballet, are submitted to regular flexibility training [12]. 

Various training modes, such as resistance and eccentric training, provide the mechanical stimulus to induce morphological changes in the muscle-tendon unit by altering fascicle length, muscle thickness and volume, and pennation angle [13,14]. The effect of these types of training on muscle structure is well documented however, evidence is limited on stretching interventions [15], although flexibility is considered a major component of physical fitness [16]. Long-term static stretching interventions in humans, examining differences in muscle architecture and joint range of motion (ROM), presented equivocal results [17,18]. For example, Freitas and Mil-Homens [17] found a significant increase in biceps femoris fascicle length (+12.3 mm, *p* = 0.04) in physically active participants, following 8 weeks of intensive static stretching training (450 sec of stretching repeated 3 times per week). In contrast, Lima et al. [18] did not observe any changes in biceps femoris and vastus lateralis muscles architecture after 8 weeks of training in 12 physically active participants, using a short-duration, static stretching (3 sets of 30 sec of stretching, 3 times per week). The discrepant results between studies may be due to the different stretching training volume and intensity, combined with the short duration of the interventions (~8 weeks) [15]. Along this line, recent cross-sectional studies that examined populations with a chronic flexibility training background (>15 years of systematic stretching) reported that professional ballet dancers [11] and elite level rhythmic gymnasts [19] had longer resting fascicle length in gastrocnemius medialis compared to controls or not trained in flexibility athletes. These studies highlight that muscle architecture differs between athletes with different flexibility-training history; although sport-specific selection criteria or heredity may also be reflected in the dissimilarities in muscle architecture observed. Therefore, examining differences in muscle architecture in youth athletes submitted to different training load characteristics, provides useful information on muscle longitudinal growth in typically developing children and allows for the definition of exercise prescription in clinical populations. To this end, this study examined differences in gastrocnemius medialis (GM) architectural properties at rest and during stretching between child rhythmic gymnasts who trained and competed for at least 2 years, with same age girls participating in volleyball training. Rhythmic gymnasts were selected for their large joint ROM and compliant muscles and also due to their extensive flexibility training [20] while volleyball players were selected because their training included much less stretching training volume [21]. It was hypothesized that flexibility trained child female athletes would have longer fascicles at rest and during stretching compared to same age, flexibility not trained athletes.

## 2. Materials and Methods

### 2.1. Subjects

Ten female rhythmic gymnasts and six volleyball players, aged 8–10 years, took part in this study. Rhythmic gymnasts who competed at Hellenic, age-group (8–10 years old) all-around competitions were recruited from different gymnastics clubs and represented the flexibility trained group (FT). Volleyball players were participating in volleyball training in one club and were considered as not trained in flexibility athletes (FNT). Both sports, rhythmic gymnastics and volleyball, involve weight-bearing activities, but, rhythmic gymnastics training includes systematic stretching (≈40–60 min per session), while volleyball training includes <10 min of stretching exercises per session [22,23,24,25]. Athletes’ characteristics are shown in Table 1. Maturity offset was calculated according to Mirwald et al. [26]. Before participating in the study, the athletes and their parents were informed about the aim and procedures of the study and provided written informed consent. The athletes had no injuries of the lower limbs for the past six months. The study design and procedures were in accordance with the declaration of Helsinki. The Institutional Ethics Committee approved the study (registration number: 1040, 14 February 2018). 

### 2.2. Experimental Design

In order to examine differences in gastrocnemius medialis (GM) architectural properties and ankle angle between child athletes with different flexibility training history, all participants were tested over two sessions. In the first, familiarization session, athletes became familiar with the study protocol. Anthropometric characteristics of the athletes were also assessed during this session. In the main testing session, athletes’ ankle angle and GM architectural characteristics were assessed in two conditions: (a) at rest and (d) during stretching. Resting ankle joint angle and GM architecture (fascicle length, pennation angle, muscle thickness) were assessed with the athletes lying in prone position on a physiotherapy bed for 20 min. (for detailed information, see description below). Following measurements of ankle angle and GM architecture at rest, athletes were standing for two min. Then, athletes performed a 1-min standing ankle dorsiflexion. Five seconds before the end of the stretching intervention a pause was imposed to obtain still ultrasound images. At the end of the 1 min stretching maximal ankle dorsiflexion was also assessed (for detailed information, see description below). Νo intense exercise or stretching was allowed in the 48 h preceding testing.

#### Anthropometric Characteristics

Height was measured without shoes with the use of a stadiometer, and body mass was measured with a calibrated digital scale (Seca 208 and Seca 710, Hamburg, Germany). Body mass index was calculated as the ratio of body weight to the squared standing height (kg/m^2^). The length of each lower extremity was measured as the distance between trochanter major to the floor with the participants in standing position. The distance between tibiofemoral joint cleft and medial malleolus was determined as calf length.

### 2.3. Gastrocnemius Medialis Architecture and Ankle Joint Angle at Rest

In order to avoid trigonometric estimations or multiple scans along the muscle length to be assembled [27], in the present study, panoramic ultrasound images were obtained, via extended-field-of view imaging, along the fascicle length of GM.

**Figure 1:**
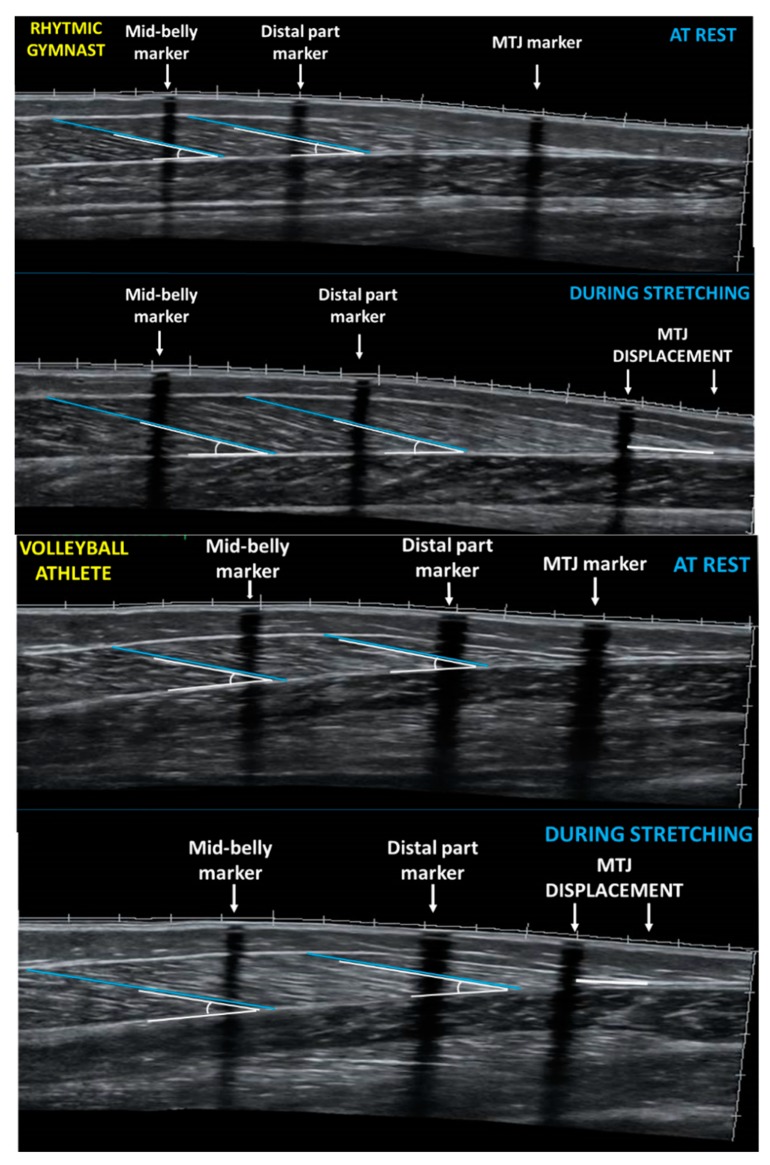
Panoramic sonographic image of gastrocnemius medialis of a rhythmic gymnast (top panel) and a volleyball player (bottom panel) at rest and during stretching showing fascicle length and pennation angle at the mid-belly and at the distal part of GM. MTJ: muscle-tendon junction.

All ultrasound measurements were performed in the morning and after athletes remained in a prone position on the examination bed, with their ankles hanging loosely on the outside of the bed for at least 20 min [28]. Muscle architecture of the right leg GM, (dominant leg that is in stance while kicking a ball) was obtained with a 10 MHz linear probe (38 mm) via extended field of view mode (Product model Z5, Shenzhen, Mindray Bio-Medical Electronics Co., Ltd., Shenzhen, China). Ultrasound images were recorded at the medial and the distal part of the GM muscle belly: one-third and half of the distance from the popliteal crease to the center of the medial malleolus, respectively. These points were marked on the skin using an echo-absorptive tape that served as reference marker [28] (Figure 1). In order to measure musculotendinous junction (MTJ) displacement, MTJ was located by real-time static ultrasound imaging and marked on the skin by an echo-absorptive tape, as well. The transducer was orientated perpendicular to the skin and parallel to the fascicles to minimize perspective and parallax measurement errors [29]. A probe path (dashed line) was drawn on the skin with a permanent pen by using static ultrasound according to the fascicle path seen from the ultrasound image. A single view was taken by moving continuously the probe in a slow and steady rate along the marked path. For each part of the muscle (medial and distal), three different fascicle lengths were measured from the deep aponeurosis to the superficial aponeurosis with a linear trace. Where the muscle fascicles met the lower aponeurosis the respective pennation angles were measured. The average of the lengths and angles of the three fascicles was used for statistical analysis for each part of the GM. The distance between the superficial and deep aponeuroses was determined as muscle thickness. Two consecutive measurements for each part of the muscle were assessed, and the average value was used for further analysis. All images were analyzed with image analysis software (Motic Images Plus 2.0, Motic, Hong Kong, China). Test-retest reliability was determined by using the intraclass correlation coefficient on 6 participants, on two separate days. The ICC (two-way random effects) for muscle fascicle length was 0.93 (95% CI: 0.576–0.990, *p* = 0.000), for muscular thickness it was 0.90 (95% CI: 0.474–0.984, *p* = 0.001), and for pennation angle, 0.95 (95% CI: 0.689–0.993, *p* = 0.001).

Ankle joint angle at rest was also measured with the athletes lying in a prone position, with their ankles hanging loosely off the bed. Resting ankle joint angle was defined as the angle created by the intersection of the femur-tibia to lateral malleolus line and lateral malleolus to fifth metatarsal line [19]. Reflective markers were placed on these anatomical points in order to define the ankle angle using a digital camera (Casio Exilim Pro EX-F1, Shibuya, Tokyo, Japan). Image analysis was performed via a free software (Tracker 4.91© 2016 Douglas Brown). Intra-class correlation coefficients for resting ankle angle was 0.98 (95% Confidence Intervals (CI): 0.833–0.998, *p* = 0.000).

### 2.4. Gastrocnemius Medialis Architecture and Ankle Joint Angle during Ankle Dorsiflexion Stretching

Panoramic ultrasound images from the two parts of the GM muscle belly (medial and distal) were obtained following the method described above. Reflective motion analysis markers, and echo-absorptive tapes remained on the skin, and the drawn path (dashed line) of the resting measurements was used, to re-assess the regions of interest. Following two minutes of standing, all athletes performed a slow, passive standing dorsiflexion stretching, for one minute. Five seconds before the end of the stretching intervention a pause was imposed to capture still images. To obtain GM ultrasound images during stretching, the probe was placed 38 mm above the skin marker that identified the middle part of the muscle belly. In addition, ‘MTJ displacement’ was defined as the difference between the MTJ marker at rest and MTJ point during stretching (Figure 1).

Ankle dorsiflexion stretching while standing is commonly performed in sport practice [30], and the athletes were familiar with it. Stretching was performed with the athletes barefoot. Athletes were instructed to relax while they passively stretched their ankle plantar flexors, in a slow and continuous manner. The foot to be tested (right) was placed on the midline of a marked area on the floor, and the left foot was placed forward at step-length distance. The end point of standing dorsiflexion stretching was defined as the point that the athletes felt discomfort without lifting their heel and with no pelvic rotation. The athletes put their hands against the wall to maintain balance and were asked to keep the extended position of their hip and knee joints, during stretching [30]. Stretch intensity was indicated by the athletes using the 0–10 Wong-Baker FACES Pain Scale for children [31] to ensure that stretch achieved the point of discomfort (~8 on a scale of 0–10). During the execution of the stretch, participants were instructed to reach a pain of discomfort level of 8 in the scale of 0–10, and thus they held the stretch at exactly this perceived intensity. The psychometric properties of this commonly used pictorial scale assessing acute pain have been found to be appropriate for children over the age of 3 [31]. Six faces depict different expressions, ranging from “no hurt” to “extremely upset from pain”. A digital camera (Casio Exilim Pro EX-F1, Shibuya, Tokyo, Japan) was placed perpendicular to the plane of motion of the right leg in order to record the standing ankle dorsiflexion angle. Stretching ankle joint angle was analyzed using reflective markers placed on the knee, ankle and fifth metatarsal and calculated using free software (Tracker 4.91© 2016 Douglas Brown). Maximal standing dorsiflexion was defined as the intersection of a line joining the knee and ankle markers and horizontal (a line crossing the heel and the fifth metatarsal).

### 2.5. Statistical Procedures

Descriptive statistics were calculated. Shapiro-Wilks test checked for normality of data distribution. Pearson correlations coefficient (*r*) detected linear relations between the examined variables. Unpaired T-test examined differences between groups in anthropometry and architectural characteristics of GM at rest. A two-way ANOVA (time x group) with repeated measures for time (rest or stretch) and group (flexibility trained vs. not trained) was conducted separately for the medial and the distal part of the muscle, to examine the effect of stretching on fascicle lengths, pennation angles and thicknesses, and ankle joint angle. A Tukey post-hoc test was performed when a significant main effect or interaction was observed (*p* < 0.05). Effect sizes calculation for pairwise comparisons was performed with Cohen’s *d* [32]. To assess test-retest reliability the intra-class correlation coefficients (ICCs) were used. Statistical significance was set at *p* < 0.05. Statistical analyzes were conducted using SPSS (SPSS Statistics Version 25.0, IBM corporation, Armonk, NY, USA).

## 3. Results

### 3.1. Gastrocnemius Medialis Architecture and Ankle Joint Angle at Rest

Resting fascicle length of FT athletes was similar to FNT at the mid-belly (4.19 ± 0.37 vs. 4.24 ± 0.54 cm, respectively, t_14_ = −0.204, *p* = 0.841) and the distal part of gastrocnemius medialis (4.25 ± 0.35 vs. 4.18 ± 0.65 cm, respectively, t_14_ = 0.284, *p* = 0.780). FT and FNT athletes displayed also similar pennation angle and muscle thickness at the mid-belly (t_14_ = 0.661, *p* = 0.519 and t_14_ = 0.002, *p* = 0.998, respectively) and the distal part of the gastrocnemius medialis (t_14_ = −1.297, *p* = 0.216 and t_14_ = 0.807, *p* = 0.433, respectively) (Table 2). Resting ankle angle was larger in FT by 8% compared with FNT (120.86 ± 4.19° vs. 110.95 ± 5.79°, respectively, t_14_ = 3.982, *p* = 0.001) (Table 2).

### 3.2. Gastrocnemius Medialis Architecture and Ankle Joint Angle during Ankle Dorsiflexion Stretching

During stretching, the elongation of fascicles was greater in FT athletes compared to the FNT at the mid-belly of the muscle by 23% (+1.67 ± 0.37 cm vs. +1.28 ± 0.22 cm, *p* = 0.048) and the distal part by 47% (+1.84 ± 0.67 vs. +0.97 ± 0.29 cm, *p* = 0.013). Furthermore, FT athletes displayed greater maximal ankle dorsiflexion by 13% (*p* < 0.001), as well as greater muscle tendon junction displacement by 33% (*p* < 0.001) (Table 2). No differences were found between groups in muscle thickness at mid-belly and at the distal part (*p* > 0.053). However, FNT athletes displayed greater pennation angle at the mid-belly of gastrocnemius medialis (−2.90 ± 1.29 vs. −4.93 ± 1.91 vs., *p* = 0.048), but not at the distal part (*p* = 0.362) (Table 2).

### 3.3. Correlations Between Fascicle Length, Ankle Angles and MTJ Displacement

When all athletes were considered as a group, significant correlations were found between fascicle elongation at the distal part of GM and MTJ displacement (*r* = 0.752, *p* < 0.01) and ankle angle during stretching (*r* =−0.638, *p* < 0.01). Moreover, a significant correlation was found between MTJ displacement and ankle angle during stretching (*r* = −0.610, *p* < 0.05).

## 4. Discussion

This study examined differences in GM architectural properties at the middle and the distal part of the muscle belly, at rest and during stretching, between flexibility trained and not trained female athletes (rhythmic gymnasts and volleyball players, respectively), aged 8–10 years. The main finding of this study was that, at rest, the two groups displayed similar GM architectural properties, but during stretching FT displayed greater fascicle elongation at the middle and the distal part of GM, and greater MTJ displacement. In addition, FT had larger ankle joint angles at rest and larger change in ankle angle during stretching, compared with FNT, athletes. Significant correlations were found between fascicle elongation at the distal part of GM, MTJ displacement and ankle angle during dorsiflexion.

Gastrocnemius muscle is a prime mover in ankle plantar flexion and thus its architecture is related to force/power production and range of motion [5]. However, chronic modifications to gastrocnemius muscles architecture because of exercise or training in children are currently unknown [33,34,35]. The results of this study indicated that the two groups had similar resting fascicle length at the medial and the distal part of GM. This finding is interesting because recent cross-sectional studies found longer resting fascicle length in flexibility trained, compared with untrained adult participants. For example, a previous study [11] compared professional ballet dancers to controls and found that ballet dancers had longer fascicles in GM in resting prone position (55 ± 5 vs. 47 ± 6 mm, respectively). Another study that examined elite rhythmic gymnasts and female volleyball players, also reported that gymnasts had longer fascicle length at rest at the mid-belly and the distal part of GM compared to volleyball players, by 20 and 18%, respectively [19]. Resting fascicle length has been recently linked with plantar flexion torque and work in healthy adults [34] and was related to the muscle’s force-length relationship [35]. The participants of the present study were growing children, aged 8–10 years. During growth, muscle-tendon units are increased in length, to keep up with increases in bone length [10,35]. Benard et al. [10] examined how maturational growth and skeletal development imparts changes in muscle architecture and found that GM muscle length increases (through an increase in muscle, tendon and fascicle length) approximately 6% per year from age 5 to 12, in proportion with increases in tibia length. That study also reported that the length component of the physiological cross-sectional area of GM as well as muscle fascicles, increased in length [10]. Thus, even if long-term systematic and extensive flexibility training might increase muscle fascicle length, it is plausible that the mechanical stimulus of stretching training is not adequate to induce changes additional to maturational growth in developing children. Nevertheless, cross-sectional study-designs do not imply causation and, at present, the chronic effect of static stretching training on joint range of motion and muscle architecture in humans is not sufficiently documented.

In the present study, fascicle elongation was measured at mid-belly and at the distal part of GM during maximal ankle dorsiflexion. This was because the mid-belly might not accurately reflect muscle architecture across the entire gastrocnemius muscle [36]. The results of this study indicated that fascicle elongation was greater in FT athletes compared to FNT at the middle (*p* = 0.048, *d* = 1.21) and the distal part of GM, (*p* = 0.013, *d* = 1.59) by 23 and 47%, respectively. This result is in line with previous research reporting greater elongation of GM fascicles in flexible compared to inflexible subjects [19,37]. Importantly, an almost twofold greater elongation was observed in FT athletes at the distal part of GM compared with FNT (+1.84 ± 0.70 vs. 0.97 ± 0.32 cm, respectively, *p* = 0.013) and significantly greater MTJ displacement (*p* = 0.001, *d* = 2.24). Simpson et al. [38] examined adaptations in architectural characteristics of gastrocnemius medialis and lateralis following 6 weeks of stretching training, in adult participants. The authors reported that muscle fascicles in the belly increased by 5.1% by week 6 whereas fascicles in the junction were 25% longer [38]. A previous study also reported that adult rhythmic gymnasts displayed greater fascicle elongation at the distal part compared to volleyball players (45 vs. 39%, respectively, *p* = 0.026) [19]. This finding highlights that there may be non-uniform morphological adaptations along the length of a bi-articular muscle, like GM, depending on training history. Chronic flexibility training and/or other components of sport-specific training may induce muscle architectural adaptations that differ between the muscle belly and the region near the musculotendinous junction. Previous animal studies identified higher levels of myosin heavy chain mRNA at the MTJ of fibers stretched for 4 days [39] and suggested that fiber lengthening, following stretching, created a need for contractile protein synthesis and assembly into myofibrils at the MTJ [40]. A recent study in humans also reported that following a 4-weeks resistance training intervention, the remodeling of muscle fibres near the MTJ was very high [41].

Enhanced joint ROM following static stretching training has been shown with various stretching protocols in youth athletes or in physical education settings [42,43]. This study examined ankle angle at rest lying in prone position, and during maximal ankle dorsiflexion. The results of the present study indicated that at rest, rhythmic gymnasts had greater ankle angle by 8%, compared with volleyball players (*p* = 0.001, *d* = 1.21) (Table 2). A similar finding was reported in a previous study with adult rhythmic gymnasts, and the authors assumed that different resting ankle joint angle between groups may imply a different slack length in the muscles surrounding the ankle joint due to long-term, extensive flexibility training [19]. It is not known whether flexibility training and/or other components of sport-specific training may alter the “neutral”, resting ankle joint angle [11]. Previous studies in adults reported similar ankle joint angles at rest between flexibility trained and not trained subjects [11,37]; however, further research is required on the impact of chronic flexibility training on body tissues determining joints range of motions.

Ankle joint dorsiflexion angle was also significantly greater in FT compared to FNT athletes by 13% (*p* = 0.001, *d* = 2.88), and muscle tendon junction displacement by 33% (*p* < 0.001, *d* = 2.24) (Table 2). Moltubakk et al. [11] and Donti et al. [19] also found larger ankle dorsiflexion angle in adult ballet dancers and gymnasts compared to controls, a fact mirroring their regular, intensive stretching training. Acute increases in joint ROM following stretching are mainly due to an increased tolerance to stretch [44]. The association of chronic increases in joint ROM with adaptations in muscle architecture has not been clearly established [17,18,37]. Some previous long-term stretching interventions in adults, indicated enhanced joint ROM followed by concomitant increases in fascicle length [15,38] while other long-term stretching interventions failed to detect changes in muscle architecture [18,37]. Amongst the factors determining joint ROM, maximal fascicle elongation at the distal part of the muscle belly and MTJ displacement in the present study, were strongly associated with larger maximal ankle dorsiflexion angle (*r* = −0.638, *p* < 0.01, and *r* = −0.610, *p* = 0.05, respectively). However, the cross-sectional design of this study limits interpretation of these findings. In addition, available studies indicate that there is considerable variation in GM muscle architecture associated with chronological age [8]. Thus, the small number of participants in this study is a limitation that should be acknowledged. Chronic intervention studies are required in developing athletes, to distinguish genetic or acquired through years of sport-specific training changes in muscle architecture in order to examine the contribution of changes in fascicle length to the increase in muscle length in typically developing children. It should be noted that the time frame of middle childhood (6–11 years) has been proposed as a ‘window of opportunity’ for developing flexibility and as a sensitive period for morphological changes [45,46].

## 5. Conclusions

Collectively, greater muscle elongation at the mid-belly and the distal part of GM during static stretching, and greater ankle angles at rest and during dorsiflexion were observed in FT compared to FNT female athletes, aged 8–10 years. These findings indicate that between children with different flexibility training history, muscle architecture differs only during stretching, and that there are non-uniform adaptations along GM length depending on training history. Albeit speculative, increased muscle fascicle elongation may represent an early-stage adaptation to stretching-induced increases in resting fascicle length found in flexibility trained, female adult athletes compared with not trained controls.

## Figures and Tables

**Table 1:** Anthropometric characteristics of the participants (means ± standard deviation).

Anthropometric Characteristics	Flexibility Trained Athletes (n = 10)	Flexibility Untrained Athletes (n = 6)	*t* (14)	*p*
Age (y)	9.00 ± 0.56	9.00 ± 0.63	0.000	1.000
Training experience (y)	3.70 ± 1.25	2.67 ± 0.52	1.906	0.770
Height (m)	1.34 ± 0.61	1.38 ± 0.3	−1.388	0.187
Body mass (kg)	27.57 ± 3.44	40.15 ± 5.89	−5.450	0.000
Body Mass Index(kg/m^2^)	15.29 ± 1.15	21.05 ± 2.39	−6.565	0.000
Knee height (cm)	29.20 ± 2.03	30.32 ± 2.08	−1.057	0.308
Leg length (cm)	69.30 ± 3.62	70.33 ± 2.34	−0.622	0.544
Maturity offset	−5.52 ± 0.17	−4.99 ± 0.26	−5.023	0.000

**Table 2:** Changes in muscle architecture characteristics and ankle angle following stretching for the flexibility trained (FT) (n = 10) and not trained athletes (FNT) (n = 6).

Variables	Athletes	Pre−Stretching Measurements	Δ Values (Pre−vs. Stretching)	*p*	Cohens’ *d* (Pre–Post Stretching)	Cohens’ *d* of Δ Values between Groups
Fascicle Length Mid−belly (cm)	FT	4.19 ± 0.37	+1.67 ± 0.39	0.048	5.30	1.21
	FNT	4.24 ± 0.54	+1.28 ± 0.24		2.60	
Fascicle Length Distal part (cm)	FT	4.25 ± 0.35	+1.84 ± 0.70	0.013	4.58	1.59
	FNT	4.18 ± 0.65	+0.97 ± 0.32		1.62	
Thickness Mid−belly (cm)	FT	1.53 ± 0.12	+0.23 ± 0.06	0.053	1.94	1.25
	FNT	1.53 ± 0.24	+0.15 ± 0.10		0.73	
Thickness Distal part (cm)	FT	0.74 ± 0.22	+0.46 ± 0.16	0.061	2.31	1.14
	FNT	0.64 ± 0.25	+0.29 ± 0.16		1.20	
Pennation angle Mid−belly (°)	FT	21.76 ± 1.76	−4.93 ± 2.01	0.048	3.57	1.19
	FNT	21.19 ± 1.43	−2.90 ± 1.41		2.09	
Pennation angle Distal part (°)	FT	18.00 ± 1.96	−2.48 ± 2.60	0.362	1.44	0.52
	FNT	19.46 ± 2.51	−1.35 ± 1.70		0.69	
Ankle angle (°)	FT	120.9 ± 4.2	−62.7 ± 6.7	0.001	13.25	2.88
	FNT	111.0 ± 5.8†	−44.9 ± 6.3		8.92	
MTJ Displacement (cm)	FT		+2.31 ± 0.40	0.001		2.24
	FNT		+1.54 ± 0.30 ^†^			

^†^: *p* < 0.001 from the corresponding value in flexibility trained athletes.
